# Poplar Tree Response to Feeding by the Petiole Gall Aphid *Pemphigus spyrothecae* Pass

**DOI:** 10.3390/insects11050282

**Published:** 2020-05-05

**Authors:** Izabela Kot, Katarzyna Kmieć

**Affiliations:** Department of Plant Protection, Faculty of Horticulture and Landscape Architecture, University of Life Sciences in Lublin, 7 Leszczynskiego Str., 20-069 Lublin, Poland; izabela.kot@up.lublin.pl

**Keywords:** antioxidant enzymes, aphids, cytoplasmic membrane condition, galls, oxidative stress, poplar tree, ROS accumulation

## Abstract

*Pemphigus spyrothecae* Pass. which is a member of the subfamily Pemphiginae is one of the gall-inducing aphids that occurs on poplar trees. Phloem feeding of a founding mother on leaf petiole results in the formation of a new organ, i.e., the spiral gall. This study documents aphid development inside the galls during the growing season and the effect of their feeding on leaf architecture and physiology of the host plant. In particular, leaf length, width, and area were measured, as well as hydrogen peroxide (H_2_O_2_) content, electrolyte leakage (E_L_), malondialdehyde (MDA) concentration, and the activity of ascorbate (APX) and guaiacol peroxidase (GPX) were determined in galls and galled leaves. The presence of petiole galls significantly decreased the length, width, and leaf area. Aphid activity increased H_2_O_2_ concentration in galls and E_L_ from galls and leaf tissues, which was accompanied by a strong decrease in MDA content and both peroxidase activities, especially in gall tissues. It can be suggested that *P. spyrothecae* can manipulate physiological machinery of the host plant for its own benefit.

## 1. Introduction

Gall-inducing aphids have a restricted range of primary hosts, as the tribe Pemphigini parasitizes on *Populus*, Eriosomatini on *Ulmus,* and Fordini on *Pistacia* [1]. At present, 14 aphid species of the tribe Pemphigini, subfamily Pemphiginae, are known to induce conspicuous galls on poplar trees in Europe [2]. Only one of these, *Pemphigus spyrothecae* Pass., has a holocyclic life cycle and develops exclusively on the primary host poplar trees. Other Pemphigus species alternate between *Populus* and roots of different dicotyledons [3]. The trees of the genus *Populus* (Salicaceae) are an important component of natural and urban habitats. This is related to their wide geographic distribution, which gives rise to a great number of varieties and cultivars, rapid growth in various challenging conditions, and tolerance to a wide range of soils or disturbed habitats [4].

*P. spyrothecae* has the following four generations during the life cycle: fundatrix (stem mother), apterous virginoparae (virgin producer), alate sexuparae (sexual morph producer), and sexuales [5]. It feeds near the center of leaf petioles of *Populus nigra* L., especially on its variety “Italica”. It induces a spiral-shaped gall (usually consisting of three spirals) that completely surrounds fundatrix and its offspring [6]. Galls of *P. spyrothecae* are induced only by a fundatrix which is an extremely specialized and conservative stage [7]. A gall founder appears from the fertilized egg in spring, induces a gall on the petiole of the host plant, and produces offspring parthenogenetically and viviparously in the gall. These galls are associated with the evolution of social behavior and the appearance of soldiers in the virgin population. Usually, about half of the aphids in a gall are soldiers [8]. The function of the soldier caste is to defend a colony against predators, colony hygiene, and nest repair [9]. The offspring of fundatrices mature into apterous virginoparae, and these produce the third generation which all become alate sexuparae. Mature sexuparae leave galls and produce a small number of males and females on the bark of poplar trees that subsequently mate. Fertilized females always lay one egg covered with wax fibers in bark crevices of the host tree [5,10].

Chemicals responsible for gall induction are called “cecidogenic substances”. However, aphids feed directly on phloem sap, diverting plant assimilates to the galls. Therefore, to induce the growth of the gall, they require internal stimulation of cell growth and division, thereby regulating the size and shape of the forming galls [11]. They also affect host a defense response. The earliest response of plants to insect feeding is often associated with protein phosphorylation, membrane depolarization, calcium influx, and increased production of reactive oxygen species (ROS), including free radicals (OH^•^), (O_2_^•^), (HO_2_^•^), and (RO^•^), as well as non-radical forms (H_2_O_2_) and (^1^O_2_) [12]. The ROS production in gall tissues occurs during the entire process of gall formation and the peak is observed during its maturation [13]. Each ROS differs in biochemical properties, although its generation in plant tissues after insect feeding affects membranes, biomolecules, nutrient concentration, and enzymatic activities. During biotic stress, the H_2_O_2_ concentration in plant tissues is the highest as compared with other reactive oxygen species, thus, significant attention has been paid to it in recent studies [14]. H_2_O_2_ reacts with all types of organic molecules and easily diffuses across cell membranes [15]; it is also involved in the initiation of lipid peroxidation (LPO) in biomembranes [16]. LPO is known as an informative and diagnostic tool that evaluates membrane integrity due to its effect on biological membrane structures, their low fluidity and higher permeability, followed by increasing electrolyte leakage and decreasing ion percentage caused by a loss of enzymatic activity [17]. LPO can be determined by measuring secondary products such as malondialdehyde (MDA) and other thiobarbituric acid reactive substances accumulation. These products are accepted markers of oxidative stress in plants [18].

Enzymatic (e.g., peroxidases, dismutase, and catalase) and non-enzymatic (e.g., ascorbic acid, glutathione, tocopherol, and carotenoids) components are considered to maintain low cellular ROS concentrations. They can stimulate the transport of insect resistance signals and respond in concert by converting reactive oxygen species into less toxic products in cells [19]. Among enzymatic antioxidants, peroxidases stand out from many other peroxide metabolizing enzymes for their high H_2_O_2_ specificity [20]. Peroxidases take part in scavenging phospholipid hydroperoxides, thereby protecting cell membranes against peroxidative damage; they can also be involved in redox transduction under stressful conditions [21]. Furthermore, guaiacol peroxidase (GPX) is involved in auxin metabolism, cell wall elongation, phytoalexin synthesis, and protection against pathogens by switching the hypersensitive response [22]. Ascorbate peroxidase (APX) is a key enzyme of the ascorbate-glutathione (AsA-GSH) cycle and also scavenges hydrogen peroxide and other ROS products [23,24]. APX is responsible for H_2_O_2_ removal from the plant cells cytosol by catalyzing its reduction to water [17,25].

*P. spyrothecae* has been the subject of ecological studies [5,10,26], experimental evidence regarding soldier behavior [27,28,29,30], colony defense against attacks by natural enemies [5,31], effects on leaf chemistry [32,33], and foliage photosynthetic characteristics [34], during the last decades. However, no studies have specifically focused on the host plant defense responses to the feeding of this species. Therefore, the current study was conducted to test aphid reproduction and feeding in order to understand host plant manipulation and its response to gall induction. We tested the hypothesis that H_2_O_2_ and MDA contents, as well as GPX and APX activities, as part of plant defense responses, would change in the same general direction (i.e., increase or decrease) when leaves are subjected to stress. It is known that gall-inducing species can suppress the defensive response in host plants for their own needs.

## 2. Materials and Methods

### 1.1. Study Site and Sampling

The study was carried out in Lublin (Poland) (22°34′ E, 51°14′ N) during the 2016–2017 growing season. Plant material was collected from the end of April until the end of October from trees of *Populus nigra* “Italica”. Individual poplar trees, which were part of urban green areas, were marked (n = 10) and subsamples of 10 leaves with galls were randomly collected from each tree at 10-day intervals using the visual method. One sample consisted of 100 leaves collected from trees within the reach of the hand. Leaves were cut off with scissors and brought in plastic bags to the laboratory within 1 h after collection. In the laboratory (Department of Plant Protection, Subdepartment of Entomology, University of Life Science in Lublin, Lublin, Poland), individual galls were cut open with a scalpel and aphids were gently transferred to a Petri dish. Moults and honeydew were separated with a brush, and live developmental stages of aphids and their number were assessed under a microscope.

Additional samples of 100 phenologically similar galled and non-galled leaves were collected from marked trees, during the end of July each year of the study, to verify the influence of gall induction on leaf area and the impact of aphid number on gall size. The length, width, and surface area of non-galled and galled leaves were measured in the laboratory using a CI-202 Laser area meter (CID, Camas, WA, USA). Aphid counting in galls was preceded by measuring gall length and width with a DIN863 Digital Micrometer (MIB, Messzeuge, Spangenberg, Germany).

### 1.2. Laboratory Assay

#### 1.2.1. Plant Material for Physiological Analyses

In June, when galls were fully developed, an additional 50 leaves with galls and 50 without galls were randomly taken from marked trees. In the laboratory, leaves with galls were dissected to separate aphids and plant tissue, while leaves without galls were used as the control. The plant material was categorized as follows: (1) control (leaves without galls), (2) leaves on which the galls were present, (3) galls. Such samples were weighed and used directly to analyze the activity of the defense enzymes such as GPX and APX, hydrogen peroxide (H_2_O_2_), and malondialdehyde (MDA) contents, as well as E_L_. The analyses were carried out in 3 replications.

#### 1.2.2. H_2_O_2_ Content

The H_2_O_2_ content was estimated following the methods employed by Jena and Houdhuri [35]. Fresh plant material (0.5 g) was ground in 3 mL of phosphorus buffer (50 mM, pH 6.5) at 4 °C. Then, the homogenate was centrifuged at 6000× *g* for 25 min. Next, 1.5 mL of the supernatant was added to 0.5 mL TiO_2_ in 20% (*v*/*v*) H_2_SO_4_ and centrifuged again at 6000× *g* for 15 min at room temperature. The absorbance was read at 410 nm using a spectrophotometer (CE 9500, Cecil, Cambridge, UK). The H_2_O_2_ content was calculated using the molar absorbance coefficient of 0.28 μM^−1^ cm^−1^ and expressed as nanomoles per 1 g fresh weight.

#### 1.2.3. Electrolyte Leakage (E_L_) Assay

The electrolyte leakage (E_L_) assay was determined by following the method of Kościelniak [36] using an Elmetron CC-317 microcomputer conductometer. Ten leaf rings of 0.9 cm diameter were cut with a cork borer from each sample. Then, plant material was subsequently transferred to 20 cm^3^ of deionized water and incubated at room temperature on a rotary shaker for 24 h. Then, the initial electrical conductivity (K1) was measured. The samples were autoclaved at 100 °C for 15 min and final conductivity (K2) of the solution was measured after 24 h of shaking. Electrolyte leakage was calculated using the following formula: E_L_ (%) = (K1/K2) × 100.

#### 1.2.4. Malondialdehyde (MDA) Content

The level of lipid peroxidation was determined as the amount of malondialdehyde content (MDA), as described by Heath and Packer [37]. Plant tissues (0.2 g) were homogenized in 0.1 M potassium phosphate buffer (pH 7.0). The homogenate was centrifuged at 12,000× *g* for 20 min at room temperature, and then 0.5 cm^3^ of supernatant was added to 2 cm^3^ of 20% trichloroacetic acid (TCA) containing 0.5% thiobarbituric acid (TBA). The mixture was incubated at 95 °C for 30 min, and then the reaction was stopped by cooling the tubes in an ice water bath. Another centrifugation was carried out at 10,000× *g* for 10 min. The absorbance was read at 532 and 600 nm using a spectrophotometer (Cecil CE 9500, UK). The concentration of MDA was calculated using the molar absorbance coefficient (155 nM^−1^ cm^−1^) and expressed as nanomoles per 1 g fresh weight.

#### 1.2.5. Peroxidase Activity

For guaiacol peroxidase (GPX, EC 1.11.1.7) and ascorbate peroxidase (APX, EC 1.11.1.11) activities, plant tissues (0.2 g) were ground in a mortar with 0.05 mol dm^−3^ phosphate buffer (pH 7.0) containing 0.2 mol dm^−3^ EDTA and 2% PVP at 4 °C. Then, the reaction mixture was centrifuged for 10 min (10,000× *g*, 4 °C) and immediately used for analysis. The GPX activity was assayed as described by Małolepsza et al. [38]. The reaction mixture contained 0.5 cm^3^ of 0.05 mol dm^−3^ phosphate buffer (pH 5.6), 0.5 cm^3^ of 0.02 mol dm^−3^ guaiacol, 0.5 cm^3^ of 0.06 mol dm^−3^ H_2_O_2_, and 0.5 cm^3^ of enzyme extract. The variation in absorbance was measured at 480 nm for 4 min, at 1 min intervals using a spectrophotometer (Cecil CE 9500, UK). The GPX activity was calculated using the absorbance coefficient for this enzyme (26.6 mM cm^−1^) and expressed as the change in peroxidase activity per fresh weight (U mg^−1^ FW).

The APX activity was estimated as per the method of Nakano and Asada [39]. The assay mixture contained 1.8 mL 0.1 M phosphorus buffer (pH 6.0), 20 μL of 5 mM sodium ascorbate, 100 μL of 1 mM H_2_O_2_, and 100 μL of enzymatic extract. The absorbance was recorded at 290 nm for 5 min, at 1 min intervals with spectrophotometer (Cecil CE 9500, UK). The activity of APX was calculated using the absorbance coefficient (2800 M^−1^ cm^−1^) and expressed as the change of peroxidase activity per fresh weight (U mg^−1^ FW).

### 1.3. Statistical Analyses

All statistical analyses were performed using Statistica 13.1 (StatSoft, Kraków, Poland). The acceptance level of statistical significance was α = 0.05. The Shapiro–Wilk test was used to asses normality. Data of physiological analyses and metric parameters of leaves are presented as arithmetic means with standard deviation (±SD). One-way ANOVA was used to distinguish the effect of *P. spyrothecae* galling activity on the following variables: H_2_O_2_, MDA, E_L_, GPX, APX in galls and leaves with galls. Means were separated by Tukey’s HSD test. The non-parametric Mann–Whitney *U* test was applied to compare the width, length, and surface area of intact leaves and leaves with galls.

## 3. Results

### 3.1. Aphid Life Cycle and Impact on Host Plant Architecture

**Figure 1:**
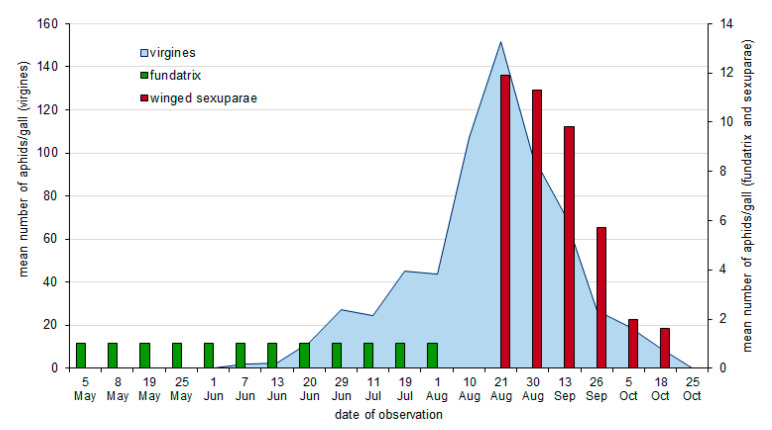
The mean number of aphids of *Pemphigus spyrothecae* in galls, in 2016.

**Figure 2:**
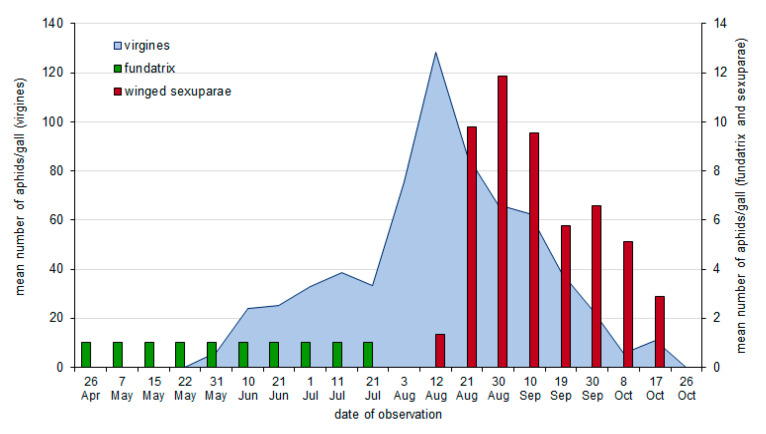
The mean number of aphids of *Pemphigus spyrothecae* in galls, in 2017.

Closure of the forming galls on poplar petioles was observed at the end of April and beginning of May (Figure 1 and Figure 2). The first fundatrix matured at the end of May, after about four weeks of feeding. The first individuals of the second generation occurred just after reaching maturity, i.e., at the end of May (2017) or at the beginning of June (2016). The offspring of fundatrices matured to apterous females in late July and in August, and began to parthenogenetically produce progeny, which developed into winged sexuparae. In 2016, the peak of aphid numbers in galls (151.8 aphids/gall on average) was observed in the third decade of August (Figure 1), while in 2017, it was observed about two weeks earlier (128.57 aphids/gall on average) (Figure 2). First winged sexuparae occurred in galls from mid-August, and they were most numerous at the end of the month. In 2016 and 2017, the highest mean number of winged aphids was similar and equaled 11.87 and 11.9 winged aphids/gall, respectively. They were observed in galls until the end of October.

The feeding activity of *P. spyrothecae* resulted in a successive enlargement of galls, which was proportional to aphid development. Table 1 shows that the average number of aphids in galls was dependent on gall size (from an average of 2.36 aphids in galls less than 10 mm long to more than 50 aphids in galls more than 20 mm long). A large, sudden increase in the number of aphids was observed in galls of 14–20 mm in length and 9–10 mm in width.

There were significant differences in leaf surface area (U = 431.00, *p* = 0.0497), leaf length (U = 394.50, *p* = 0.01640), and leaf width (U = 419.50, *p* = 0.0357) between intact leaves and leaves with galls (Table 2), indicating that the development of host leaves can be affected by the galling activity of aphids.

### 3.2. Physiological Reaction of Populus Nigra “Italica” to Galling Activity

**Figure 3:**
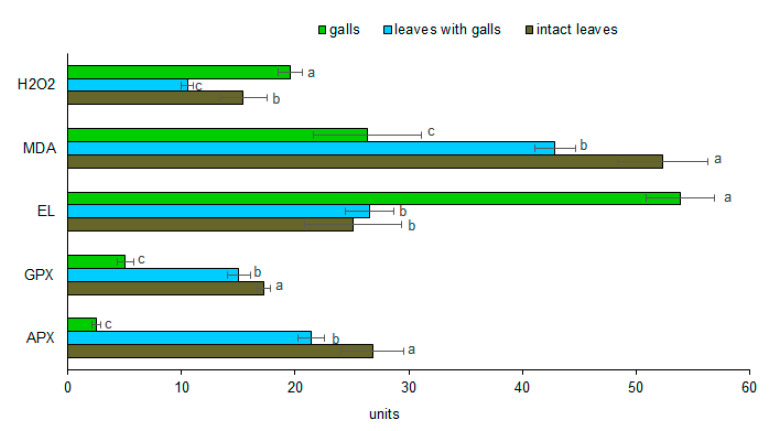
The effect of *P. spyrothecae* feeding on H_2_O_2_ (µmol g^−1^ FW) and malondialdehyde (MDA) (nmol g^−1^ FW) concentrations, electrolyte leakage (E_L_) (%) level, and activity of guaiacol peroxidase (GPX) (U mg^−1^ FW) and ascorbate peroxidase (APX) (U mg^−1^ FW) in *Populus nigra* “Italica” tissues (means ± SD). Bars sharing the same letter for each parameter do not differ significantly at *p* ≥ 0.05 (Tukey’s HSD test).

*P. spyrothecae* galling activity resulted in significant alterations in the content/activity of all analyzed physiological parameters in galls and leaves with galls on petioles as compared with intact leaves. Insect feeding had a significant effect on H_2_O_2_ concentration in all samples (F_(2,6)_ = 30.1258, *p* ˂ 0.001). The H_2_O_2_ content was significantly elevated in gall tissues, while significantly decreased in galled leaves as compared with intact leaves. The level of this free radical was almost 1.9-fold higher in galls than in galled leaves (Figure 3).

The MDA content in all three samples was significantly different from each other (F_(2,6)_ = 37.3119, *p* ˂ 0.001). A clear decrease was observed in the malondialdehyde level in tissues exposed to galling. The lowest concentration of MDA was recorded in galls, and it was almost two-fold lower than in intact leaves. Cell membrane damage, measured as E_L_ from cells, was significantly affected by aphid feeding (F_(2,6)_ = 74.2406, *p* ˂ 0.001). Electrolyte leakage was similar in intact and galled leaves, and was the highest in the galls, as a two-fold difference between gall tissues and control was measured (Figure 3).

Feeding of *P. spyrothecae* also significantly altered the activity of guaiacol peroxidase (F_(2,6)_ = 190.6116, *p* ˂ 0.001) and ascorbate peroxidase (F_(2,6)_ = 161.7282, *p* ˂ 0.001). Both antioxidant enzymes showed similar significant reduction in galled leaves and in gall tissues as compared with the control (Figure 3). Extremely low activity of GPX (5.06 U mg^−1^ FW) and APX (2.51 U mg^−1^ FW) was recorded in galls as compared with intact leaves (17.21 U mg^−1^ FW and 26.81 U mg^−1^ FW, respectively).

## 4. Discussion

Among gall-forming insects, aphids are unique, as they have evolved complex life cycles and are often host-specific insects, which is also the case with *P. spyrothecae*. Our results concerning the seasonal life history of this species demonstrate that its galling process on *P. nigra* “Italica” lasted six months. Population studies showed that the initiation of infestation occurred in April, reaching a population peak in August. From early August to the end of October, winged sexuparae emerged from the galls through the furrow between two coils. These results were found to be consistent with the findings of Urban [5]. However, this author pointed out that there were, on average, 500 *P. spyrothecae* aphids in galls with intact development in the second half of August and at the beginning of September. There were even more than 1800 aphids in some of the examined galls. According to our findings, the average number of aphids was about 150, and the maximum number in one gall did not exceed 260. A similar number of aphids in galls was observed by Alton in England [40].

Due to the mass occurrence of *P. spyrothecae* on one tree, stem mother settlement is important in terms of increased fitness. The fundatrix exhibits high activity in the search for a feeding place. According to Alton [40], it spends up to 51 min visiting leaves and petioles on the shoot, followed by probing for up to 3.5 h, and some aphids die before gall formation. Fundatrix most frequently locates on larger shoots to achieve better fitness. Aphid galls are mostly placed on the petioles of large leaves resulting in an increase in fitness as compared with galls on smaller leaves; the galls are positioned on the petiole in such a way as to maximize nutrient uptake from both the galled leaf and surrounding leaves. Larson and Whitham [41] have shown that the galls of *Pemphigus betae* Doane, located at the most basal site of the leaf, were strong sinks for photoassimilates produced in neighboring leaves. Some studies indicated that the number of offspring produced by the stem mother in the gall was positively correlated with gall volume [42,43,44], which implied that gall volume could be used as an indicator of fundatrix fitness. Our results also showed that aphid feeding during gall formation in the season led to a significant reduction in leaf area, up to 16% as compared with the control. This effect can result from drainage of assimilates towards aphid galls or inhibited biosynthetic pathways caused by aphid feeding [45].

Gall-inducing insects feed in a favorable microhabitat, on more nutritious and less protected plant tissues [46]. Nevertheless, their feeding, as a biotic stress, induces defense responses in plants that can be suppressed in the case of specialized herbivores. Plant defense strategies against herbivores comprise a wide range of physiological and biochemical mechanisms, for example, reduction of epicuticular waxes, dry weight or amino acid and sugar contents [45]. Moreover, oxidative stress, as an important part of plant response following herbivore attack, can be accompanied by an increased in ROS content and induction of certain oxidative enzymes [47].

The results of the current study showed, for the first time, ROS generation in association with changes in cell membrane stability and antioxidant enzyme activities during *P. nigra* “Italica” response to *P. spyrothecae* infestation. The production of hydrogen peroxide, as one of the reactive oxygen species, is a general response of plants to stress conditions, thus, plant cells produce H_2_O_2_ in response to aphid feeding [48,49,50]. However, Isaias et al. [13] and Isaias and Oliveira [51] have indicated that gall cells can be involved in ROS production, which in turn can trigger gall development. The current study revealed increased H_2_O_2_ levels only in gall tissues. Similarly, enhanced H_2_O_2_ production limited to the feeding site was found by Martinez de Ilarduya et al. [52] during tomato infestation by *Macrosiphum euphorbiae* (Thom.). High H_2_O_2_ concentration was also detected in mature galls of *Tetraneura ulmi* (L.) on *Ulmus pumila* L. [53], as well as galls of Psyllidae and Cecidomyiidae on Neotropical plants [13,54]. This could suggest that the defense response of the plant was locally restricted to gall tissues, whereas balance between ROS production and scavenging was preserved in galled leaves.

Oxidative stress generated during aphid feeding caused lipid peroxidation and changes in membrane permeability in *Pisum sativum* L. [55] and *Zea mays* L. [50]. In the current study, on the one hand, excessive H_2_O_2_ production and accumulation in gall tissues of *P. spyrothecae* did not increase lipid peroxidation, as measured by the MDA content. On the other hand, it significantly increased cell membrane damage in gall tissues, which was estimated by electrolyte leakage from the cells. Aphids are exclusive phloem feeders taking up nutrients while keeping phloem cells alive. They continuously inject salivary secretions into plant tissues, which prevents sieve tube occlusion and probably counteracts plant defence mechanisms [56]. Bhattacharjee [18] has suggested that low levels of membrane lipid peroxidation byproducts modulate protective cell signaling pathways, exhibiting an adaptive response.

A trend similar to the lipid peroxidation rate was found for the GPX and APX activities. These enzymes control the accumulation of H_2_O_2_ and their activity is positively correlated with the intensity and duration of stress in plant cells [57]. Kaur et al. [58] found that moderately resistant pigeon pea (*Cajanus cajan* L.) genotypes exhibited a decrease in MDA content, while moderately susceptible genotypes showed an increase in MDA content in the pod wall following *Helicoverpa armigera* (Hüb.) feeding. On the one hand, feeding of this Noctuid also resulted in a significant decline in APX activity in leaves, developing seeds, and the pod wall of intermediate and moderately susceptible genotypes. On the other hand, feeding of *Brevicoryne brassicae* L. increased the LPO rate and reduced APX activity in cabbage leaves [45]. Kmieć and co-authors [53] demonstrated an increase in ROS production associated with downregulation of antioxidant enzyme activities in mature galls and galled leaves of elm caused by *T. ulmi* galling process. Similar results regarding gall inducing Cynipidae on oak trees were obtained by Kot and Rubinowska [59]. These data have strongly suggested that plant response to insect feeding depends on insect or host plant genotype. Current knowledge on aphid saliva contents suggests that salivary secretions of galling aphids are involved in plant reprogramming and can modulate plant responses to facilitate feeding [56]. Low peroxidase activity in galls seems beneficial for insects, as these enzymes exert direct toxicity in herbivore gut and regulate the number of processes resulting in a reduction in plant digestibility [22,60].

## 5. Conclusions

In conclusion, the results presented in the current work have shown that the size of galls affect the number of *P. spyrothecae* individuals feeding inside. The presence of gall on the petiole significantly decreased the length, width, and leaf area. Galling activity of *P. spyrothecae* also affected plant physiology. Aphid feeding generated ROS overproduction and enhanced membrane permeability while decreasing MDA content, as well as APX and GPX activity in host plant tissues, especially in galls. The mode of changes suggested manipulation in plant physiology, promoting aphid feeding. It is also possible that different components of the defense mechanism coping with elevated ROS levels are involved in this insect–plant interaction system, thus, further research focused on other enzymatic or non-enzymatic scavengers is needed.

## Figures and Tables

**Table 1:** Number of *Pemphigus spyrothecae* depending on gall size.

Gall Length [mm]	Gall Width [mm]	Maximum Number of Individuals/Gall	Average Number of Individuals/Gall
<10	<5	5	2.36
10–13	5–8	7	2.87
14–20	9–10	71	27.25
>20	>10	<150	<50.00

**Table 2:** Effect of *P. spyrothecae* galls on metric parameters of *Populus nigra* “Italica” leaves.

Type of Parameter		Intact Leaves	Leaf with Gall on the Petiole
Leaf blade surface area (cm^2^)	minimal	8.12	3.56
	maximal	30.68	21.79
	mean ± SD	15.57 ± 4.77 ^a^	13.07 ± 4.65 ^b^
Leaf blade length (cm)	minimal	3.35	2.46
	maximal	6.83	6.56
	mean ± SD	4.66 ± 0.75 ^a^	4.07 ± 0.91 ^b^
Leaf blade width (cm)	minimal	3.25	1.92
	maximal	7.59	5.82
	mean ± SD	5.19 ± 0.91 ^a^	4.07 ± 0.90 ^b^

Means with the different letters in the same row are significantly different at *p* < 0.05 (Mann–Whitney *U* test).
